# On Modern Progress in Ophthalmic Medicine and Surgery

**Published:** 1894-11-17

**Authors:** Robert Brudenell Carter

**Affiliations:** Consulting Ophthalmic Surgeon to St. George's Hospital


### On Modern Progress in Ophthalmic Medicine and Surgery.

By Robert Brudenell Carter, F.R.C.S., Consulting Ophthalmic Surgeon to St. George's Hospital.

IRITIS.—(*Continued.*)

Until within a comparatively recent period in the history of medicine, the possible constitutional origin of iritis was thought to be of greater importance than its local consequences, and many observers made diligent search for indications which would furnish trustworthy evidence as to whether a given case was "syphilitic" or "rheumatic" in its character; or, if it were neither of these, from what standpoint in general pathology it should be regarded. Writers of considerable eminence laid down clear rules by which the syphilitic varieties could with certainty be distinguished from the non-syphilitic; but it unfortunately happened that these writers were seldom in complete agreement, and that the syphilitic iritis of one would have been the non-syphilitic iritis of another. Underlying the whole problem, there was the tacit assumption that "syphilitic" iritis must be treated with mercury, and that "rheumatic" iritis must be treated with alkalies and saline purgatives. Something like five-and-thirty years ago the late Mr. Zachariah Laurence declared that even syphilitic iritis was best controlled by large and repeated doses of morphia, or of some other preparation of opium; and he was accustomed to keep his patients for days in a state of semi-narcotism. I well remember his bringing a series of cases before one of the medical societies, and describing them as cases which had "recovered" from iritis under the influence of morphia. The late Mr. Dixon, in the discussion which followed the paper, expressed his approval of the word "recovered," and said that if mercury had been given, the patients would have been "cured" in half the time over which the administration of morphia had extended.

The invention and general employment of the ophthalmoscope not only served to display many conditions of the interior of the eye which had previously been concealed from observation, but it caused all the diseases of the organ to be studied and investigated with a degree of care which had never before been bestowed upon them. Among other results of this study and investigation, it became manifest to many observers that the dangers of iritis were less in the occurrence of an attack than in its tendency to recurrence, and that the tendency to recurrence was largely due to the presence of adhesions of the margin of the pupil to the surface of the anterior capsule of the crystalline lens. The eye might have recovered in all other respects, and vision might be perfect; but, if a tag of adhesion were left, this tag would constantly check the movements of the pupil in response to variations of light or to efforts at accommodation, and would keep the whole iris in a state of morbid irritability, ready to respond to any exciting cause of fresh inflammation. The establishment of this doctrine conferred upon the local consequences of iritis an importance which they had not until then been known to possess, and brought local treatment and its results into the prominence which they still retain.

In normal conditions of the eye, and in medium dilatation of the pupil, the margin of that aperture rests upon the surface of the capsule of the lens, but when the pupil is widely dilated the iris and the lens capsule are no longer in contact, being separated by a film of aqueous humour. In the ordinary, or "plastic" form of iritis, the first effect of the inflammation is to produce an effusion of sticky and tenacious lymph, by which, if the iris and the lens capsule are in contact, these structures rapidly become glued together; whereas, if the pupil is widely dilated, the lymph becomes mixed with the aqueous humour, and so diluted that its power of forming adhesions is destroyed. Very recent adhesions, moreover, may be stretched and broken by dilatation of the pupil; but if they are of more than a few hours' standing, they often refuse to yield to any dilating agent.

Even before the importance of preventing or of breaking adhesions was fully recognised, it had been discovered that belladonna was a powerful remedial agent in iritis, and it was customary to smear the brows and eyelids of the patient with the extract. A clearer understanding of the effect and value of belladonna nearly coincided with the general introduction of atropia into practice, so that the desired effect could be still better attained by a cleanly application than it had been by a very ill-smelling and disagreeable one. In a short time atropia or its sulphate came to be regarded as the sheet-anchor of treatment, and was applied to the eye in iritis in such quantities that it occasionally produced very unpleasant or even alarming symptoms. A German practitioner once went so far as to "invent" what he called an instrument for the prevention of poisoning by atropia, this instrument consisting of a sort of bulldog forceps for closing the lacrymal canals during the whole course of the treatment, in order to prevent the descent of the atropia solution through these canals into the throat. Even when used in proper moderation atropia sometimes produces dryness of the throat in susceptible people, but its employment need never be carried to the extent of doing more than this, and the German "invention" is not only exceedingly irksome or painful in use, but is also absolutely superfluous.

Apart from its value as a mydriatic, there is reason to believe that atropia exerts a powerful action through the nervous system upon the actual inflammatory process, and this action is greatly promoted by combining it with cocaine. It can no longer be regarded as an all-sufficient remedy against iritis; but it is an absolutely indispensable adjunct to other treatment, in which, in most cases, remedies that were earlier in vogue are also called upon to play their parts.

Iritis varies greatly in its more prominent symptoms. In some cases it is attended by great superficial vascularity, or by sufficient effusion or increase of secretion within the eye to occasion severe tensive pain with great impairment of vision, while in others it is insidious, and declares itself chiefly by the formation of adhesions, which are sometimes not perceived until it is almost too late to deal with them by medicinal means. The consideration of these varieties, and of the treatment they respectively require, must be reserved for the next paper of the series.

